# Defining sarcopenia: the impact of different diagnostic criteria on the prevalence of sarcopenia in a large middle aged cohort

**DOI:** 10.1007/s11357-012-9384-z

**Published:** 2012-02-08

**Authors:** A. Y. Bijlsma, C. G. M. Meskers, C. H. Y. Ling, M. Narici, S. E. Kurrle, I. D. Cameron, R. G. J. Westendorp, A. B. Maier

**Affiliations:** 1 Department of Gerontology and Geriatrics, C2-R-133, Leiden University Medical Centre, P.O. Box 9600, 2300 RC Leiden, The Netherlands; 2 Department of Rehabilitation Medicine, Leiden University Medical Centre, Leiden, The Netherlands; 3 Geriatric Department, Prince Charles Hospital, Brisbane, Australia; 4 Institute for Biomedical Research into Human Movement and Health, Manchester Metropolitan University, Manchester, UK; 5 Northern Clinical School, Faculty of Medicine, University of Sydney, Sydney, Australia; 6 Rehabilitation Studies Unit, Faculty of Medicine, University of Sydney, Sydney, Australia; 7 Netherlands Consortium for Healthy Aging, Leiden University Medical Centre, Leiden, The Netherlands

**Keywords:** Sarcopenia, Muscle, Ageing, Body composition

## Abstract

Sarcopenia, low muscle mass, is an increasing problem in our ageing society. The prevalence of sarcopenia varies extremely between elderly cohorts ranging from 7% to over 50%. Without consensus on the definition of sarcopenia, a variety of diagnostic criteria are being used. We assessed the degree of agreement between seven different diagnostic criteria for sarcopenia based on muscle mass and handgrip strength, described in literature. In this cross-sectional study, we included men (*n* = 325) and women (*n* = 329) with complete measurements of handgrip strength and body composition values as measured by bioimpedance analysis within the Leiden Longevity Study. Prevalence of sarcopenia was stratified by gender and age. In men (mean age 64.5 years), the prevalence of sarcopenia with the different diagnostic criteria ranged from 0% to 20.8% in the lowest age category (below 60 years), from 0% to 31.2% in the middle (60 to 69 years) and from 0% to 45.2% in the highest age category (above 70 years). In women (mean age 61.8 years), the prevalence of sarcopenia ranged from 0% to 15.6%, 0% to 21.8% and 0% to 25.8% in the lowest, middle and highest age category, respectively. Only one participant (0.2%) was identified having sarcopenia according to all diagnostic criteria that marked prevalence above 0%. We conclude that the prevalence of sarcopenia is highly dependent on the applied diagnostic criteria. It is necessary to reach a consensus on the definition of sarcopenia in order to make studies comparable and for implementation in clinical care.

## Introduction

Sarcopenia, low muscle mass at older age, is an increasing problem in our ageing society. Annual loss of muscle mass has been reported as 1% to 2% at the age of 50 years onwards (Buford et al. 2010; Marcell 2003), and it exceeds over 50% among those aged 80 years and older when compared to younger adults (Baumgartner et al. 1998). The change of muscle mass is closely related to changes in muscle strength. Reduced muscle strength has been found to be associated with dependency in activities of daily living (Rantanen et al. 2002; Taekema et al. 2010), cognitive decline (Alfaro-Acha et al. 2006; Burns et al. 2010; Taekema et al. 2011) and mortality (Ling et al. 2010; Cooper et al. 2010). Next to the generation of muscle strength, muscle tissue is an important reserve of body proteins and energy that can be used in extreme conditions of stress or malnutrition. Low muscle mass is associated with higher drug toxicity (Morgan and Bray 1994; Nawaratne et al. 1998) and reduced insulin sensitivity (Kalyani et al. 2012).

Since the coining of the term ‘sarcopenia’ in 1989 by Rosenberg (Rosenberg 1997), many suggestions have been made to try to establish a clinically applicable definition. In general, three possible approaches in defining sarcopenia have been suggested. According to the first, the amount of muscle mass, measured with dual-energy X-ray absorptiometry (DXA) or bioimpedance analysis (BIA), compared to a younger reference population determines whether a person has sarcopenia. Correction factors applied using this approach are height (Baumgartner et al. 1998; Janssen et al. 2004), body mass (Janssen et al. 2002), or both body height and body fat (Newman et al. 2003). In the second approach, muscle function is used as diagnostic criterion to define sarcopenia as compared to a younger reference population (Lauretani et al. 2003). The third approach combines both muscle mass and muscle function in the definition (Cruz-Jentoft et al. 2010; Muscaritoli et al. 2010).

Little is known about the degree of agreement between the diagnostic criteria and their effects on estimates of the prevalence of sarcopenia, which appears to vary extremely between different cohorts ranging from 7% to over 50% in the elderly (Abellan van Kan 2009; Baumgartner et al. 1998; Janssen et al. 2002; Lauretani et al. 2003; Newman et al. 2003). The use of different diagnostic criteria may lead to different conclusions and may have different implications for treatment. To the best of our knowledge, the differences in prevalence of sarcopenia in middle aged people comparing different diagnostic criteria have not been previously reported. In the present paper, we explore the prevalence of sarcopenia using seven different diagnostic criteria in a large cohort of Dutch middle aged people. Furthermore, we assess the degree of concordance within individuals using the different criteria. Therewith, we aim to show the importance of reaching a consensus on the definition of sarcopenia, for clinical research and patient care.

## Methods

### Study cohort

The Leiden Longevity Study (LLS) consists of long-living Caucasian siblings of 420 families together with their middle aged offspring, and the partners of the offspring as controls (Schoenmaker et al. 2006). The study included 674 participants of the middle aged to older offspring and their partners, who were assessed in the period from 2006 to 2008. The sample of partners in the study was representative of the Dutch population (Schoenmaker et al. 2006). Participants (*n* = 20) with missing data for body composition measured with Direct Segmental Multi-frequency Bioelectrical Impedance Analysis (DSM-BIA) were excluded from the present analysis. There were no selection criteria on health or demographic characteristics (Westendorp et al. 2009). The Medical Ethics Committee of the Leiden University Medical Centre approved the study, and written informed consent was obtained from all participants.

### Participant characteristics

At baseline, information on common chronic diseases and medication use was obtained from the participants’ general practitioner, pharmacist’s records and from blood sample analyses. The chronic diseases recorded were diabetes mellitus, chronic obstructive pulmonary disease, malignancy, myocardial infarction, stroke and hypertension. Health behaviour variables included current smoking status and excessive alcohol use (male >210 g/week and female >140 g/week).

### Body composition

Body mass and body height were measured. DSM-BIA was performed using the In-Body (720) body composition analyser (Biospace Co., Ltd, Seoul, Korea). We have previously shown this technique to be a valid tool for the assessment of whole body composition and segmental lean mass measurements in our sample of a middle aged people (Ling et al. 2011a). Excellent agreements were observed between the DSM-BIA technique and DXA in whole body lean mass [intraclass correlation coefficient (ICC) female = 0.95, *p* < 0.001; ICC male = 0.96, *p* < 0.001] and fat mass (ICC female = 0.97, *p* < 0.001; ICC male = 0.93, *p* < 0.001) (Ling et al. 2011a). The DSM-BIA technique is based on the assumption that the human body is composed of five interconnecting cylinders and takes direct impedance measurements from the various body compartments. A tetrapolar eight point tactile electrode system is used, which separately measures impedance of the participant’s trunk, arms and legs at six different frequencies (1 kHz, 5 kHz, 50 kHz, 250 kHz, 500 kHz, and 1,000 kHz) for each of the body segments. The spectrum of electrical frequencies is used to predict the intracellular water (ICW) and extracellular water (ECW) compartments of the total body water (TBW) in the various body segments. Lean body mass is estimated as TBW (ICW + ECW)/0.73. Body fat mass is calculated as the difference between total body mass and lean mass. The machine gives immediate detailed results including quantitative values of total body and segmental lean mass, fat mass and percentage fat mass. Appendicular lean mass (ALM) calculation was based on the sum of lean mass in all four limbs. Relative ALM was calculated as ALM divided by body height in meters squared (Baumgartner et al. 1998). Participants wore normal indoor clothing and were asked to stand barefoot on the machine platform with their arms abducted and hands gripping on to the handle of the machine.

### Handgrip strength

Handgrip strength was measured to the nearest kilogram using a Jamar hand dynamometer (Sammons Preston, Inc., Bolingbrook, IL, USA). All participants were instructed to maintain an upright standing position, arms down by the side, and holding the dynamometer in the dominant hand without squeezing the arm against the body. The width of the dynamometer’s handle was adjusted to the hand size of the participants such that the middle phalanx rested on the inner handle. Participants were allowed to perform one test trial, followed by three trials, and the best measurement was taken for analysis.

### Diagnostic criteria of sarcopenia

An overview of widely used different diagnostic criteria of sarcopenia (coded A to G), which included muscle mass and handgrip strength, is given in Table 1 (Baumgartner et al. 1998; Delmonico et al. 2007; Janssen et al. 2002, 2004; Lauretani et al. 2003). Only diagnostic criteria based on measurements of muscle mass by BIA (definition E and F) or DXA (definition A, B, C and D) scanning were used in this comparison. This overview is not a complete representation of all diagnostic criteria that have been described in the literature, for example cut-off points derived from Chinese populations are not shown (Lau et al. 2005; Woo et al. 2009). For each of the formulas described in Table 1, a different reference population had been used to derive a cut-off point for sarcopenia. For the formula ALM divided by height squared (definition A, B and C), we found three different cut-off points for men and women, established in different reference populations (Baumgartner et al. 1998; Delmonico et al. 2007; Kelly et al. 2009). Reference populations were different in age and ethnicity, consisting of younger participants of the Rosetta study (definition A) (Baumgartner et al. 1998), the NHANES survey (definition C) (Kelly et al. 2009) and the NHANES III study (definition E) (Janssen et al. 2002); elderly participants were included as reference population in the Health ABC study (definition B) (Delmonico et al. 2007) and the NHANES III study (definition F) (Janssen et al. 2004); the whole adult age range was included in the reference population in the InCHIANTI study (definition G) (Lauretani et al. 2003). The formula described in definition D was applied to our cohort, using the 20th percentile as cut-off point for sarcopenia (Delmonico et al. 2007). Consequently, we used a total of seven different diagnostic criteria of sarcopenia in our analysis.

**Table 1:** Seven different diagnostic criteria to define sarcopenia

Code	Formula	Cut-off point			Cohort used as reference population	Reference^a^
		Sarcopenia present	Men	Women		
A	ALM/height^2^	>2 SD below reference population	7.26 kg/m^2^	5.45 kg/m^2^	Rosetta Study (1986–1992), 229 non-Hispanic white men and women aged 18–40 years	Baumgartner et al. 1998
B	ALM/height^2^	Under 20th percentile	7.25 kg/m^2^	5.67 kg/m^2^	Health ABC Study (1997/1998), 2,976 men and women 70–79 years old black and white, Pittsburgh, Pennsylvania and Memphis, Tennessee	Delmonico et al. 2007
C	ALM/height^2^	>2 SD below reference population	6.19 kg/m^2^	4.73 kg/m^2^	NHANES survey (1999–2004) white men and women aged 20 years	Kelly et al. 2009
D	Residuals of linear regression of ALM with height and fat mass	Under 20th percentile	NA	NA	NA	Delmonico et al. 2007 ^b^
E (1) (2)	Skeletal lean mass/body mass × 100%	1–2 SD below reference population is class I sarcopenia >2 SD below reference population is class II sarcopenia	37% 31%	28% 22%	NHANES III (1988–1994), 6,414 men and women aged 18–39 years non-Hispanic white, non-Hispanic black and Mexican-American	Janssen et al. 2002
F (1) (2)	Skeletal lean mass/height^2^	ROC analysis was used to develop cutpoints associated with moderate (1) and high (2) physical disability	10.75 8.50 kg/m^2^	6.75 5.75 kg/m^2^	NHANES III (1988–1994), 4,502 subjects aged 60 years plus, non-Hispanic white, non-Hispanic black and Mexican-American	Janssen et al. 2004
G	Optimal cutpoint for grip strength, identified in the ROC curve, predicting walking slower than 0.8 m/s	Below optimal cutpoint	30.3 kg	19.3 kg	InCHIANTI (1998–2000), 1,030 subjects aged 20–102 years, Tuscany, Italy	Lauretani et al. 2003

*ALM* appendicular lean mass, sum measurement of lean mass in all four limbs; *ROC* receiver operating characteristics; *NA* not applicable

^a^Reference describes the formula and cut-off points, unless indicated otherwise

^b^Reference describes the formula which was applied to the Leiden Longevity Study population

### Statistical analysis

Data were analysed for men and women separately. Because there was no significant difference in fat percentage, relative ALM, and handgrip strength between offspring and partners of the LLS, data for both groups were combined (Ling et al. 2011b). The prevalence of sarcopenia in this population was assessed for all seven diagnostic criteria as described in Table 1. For definition D, the residuals of linear regression of ALM with height and fat mass were calculated.

After assigning the status of sarcopenia being present or not present in the participants according to each of the seven diagnostic criteria, participants were stratified by gender and age. The lowest age category included participants aged below 60 years, the middle age category included participants aged 60 to 69 years and the highest included participants aged 70 years and above. Differences between age groups in characteristics were assessed with linear regression or binary logistic regression. The degree of concordance within individuals using the different diagnostic criteria of sarcopenia was assessed in all participants. The difference in participant characteristics dependent on the diagnostic criterion was tested using Student’s *t*-test.

All statistical analyses were performed using SPSS for Windows (SPSS Inc., Chicago, IL, USA), version 17. *P* values <0.05 were considered statistically significant.

## Results

Baseline characteristics of the study participants stratified by gender and age are presented in Table 2. Overall, the prevalence of comorbidity was slightly higher in older participants (statistically not significant). Skeletal lean mass as a percentage of body mass and grip strength were significantly lower in the older age groups. ALM divided by height squared was significantly different between the age groups in men, but not in women.

**Table 2:** Baseline characteristics of study participants, stratified for gender and age

Variables^a^	Men				Women			
	Age (years)				Age (years)			
	≤59	60–69	≥70	*P* for trend	≤59	60–69	≥70	*P* for trend
	(*n* = 77)	(*n* = 186)	(*n* = 62)		(*n* = 128)	(*n* = 170)	(*n* = 31)	
Age (years, mean, range)	56.1 (45–59)	64.9 (60–69)	73.5 (70–82)		55.6 (38–59)	64.5 (60–69)	72.3 (70–78)	
Height (m)	1.81 (0.07)	1.78 (0.06)	1.76 (0.07)	<0.001	1.67 (0.06)	1.66 (0.1)	1.64 (0.6)	0.02
Body mass (kg)	86.7 (11.0)	85.5 (11.8)	84.1 (10.1)	0.17	71.7 (12.5)	72.6 (13.1)	76.0 (11.4)	0.15
Body mass index (kg/m^2^)	26.6 (3.3)	27.0 (3.2)	27.2 (2.9)	0.25	25.8 (4.4)	26.4 (4.7)	28.2 (4.0)	0.01
Total body fat mass (%)	23.4 (6.1)	25.8 (5.9)	28.0 (7.1)	<0.001	33.7 (7.5)	35.2 (7.1)	39.4 (6.7)	<0.001
Skeletal lean mass (kg)	37.2 (4.1)	35.3 (4.0)	33.3 (3.9)	<0.001	25.7 (3.3)	25.3 (3.3)	24.7 (3.0)	0.10
Skeletal lean mass (%)^b^	43.1 (3.5)	41.5 (3.4)	39.8 (4.0)	<0.001	36.2 (4.1)	35.3 (3.9)	32.8 (3.8)	<0.001
ALM^c^ (kg)	28.0 (3.3)	26.7 (3.2)	25.5 (3.3)	<0.001	19.3 (2.7)	18.9 (2.7)	18.9 (2.5)	0.31
Relative ALM^d^ (kg/m^2^)	8.6 (0.6)	8.4 (0.6)	8.2 (0.6)	0.002	6.9 (0.7)	6.9 (0.8)	7.0 (0.7)	0.78
Handgrip strength (kg)	51.1 (7.9)	46.8 (7.2)	41.7 (8.1)	<0.001	31.1 (6.4)	29.0 (5.0)	26.6 (4.8)	<0.001
Comorbidities (*n* %)								
Myocardial infarction	1 (1.3)	8 (4.3)	3 (4.8)	0.25	0	1 (0.6)	1 (3.2)	0.11
Stroke	1 (1.3)	5 (2.7)	5 (8.1)	0.04	2 (1.6)	3 (1.8)	0	0.70
Hypertension	10 (13.0)	50 (26.9)	20 (32.3)	0.006	25 (19.5)	47 (27.6)	13 (41.9)	0.008
Diabetes mellitus	5 (6.5)	16 (8.6)	6 (9.7)	0.48	6 (4.7)	7 (4.1)	4 (12.9)	0.25
Neoplasm	2 (2.6)	12 (6.5)	6 (9.7)	0.08	6 (4.7)	14 (8.2)	3 (9.7)	0.18
Chronic obstructive pulmonary disease	3 (3.9)	7 (3.8)	5 (8.1)	0.26	5 (3.9)	3 (1.8)	2 (6.5)	0.99
Rheumatoid arthritis	1 (1.3)	1 (0.5)	1 (1.6)	0.90	1 (0.8)	2 (1.2)	0	0.91
Sum score of medication^e^, median (IQR)	0 (0–0)	0 (0–1)	0 (0–1)	<0.001	0 (0–1)	0 (0–1)	0 (0–2)	0.02
Current smoking (*n* %)	10 (13.0)	24 (12.9)	6 (9.7)	0.61	21 (16.4)	16 (9.4)	1 (3.2)	0.02
Excessive alcohol use^f^ (*n* %)	20 (26.0)	57 (30.6)	14 (22.6)	0.95	19 (14.8)	24 (14.1)	5 (16.1)	0.90

^a^Variables are presented in mean, SD, unless indicated otherwise. *P* values for trend were calculated using linear or logistic regression

^b^Skeletal lean mass/total body mass**·**100%

^c^Appendicular lean mass, sum measurement of lean mass in all four limbs

^d^Appendicular lean mass adjusted for height (appendicular lean mass/height^2^)

^e^Sum score of total number of oral medication, data available in males (*n* = 272) and females (*n* = 268)

^f^Male >210 g/week and female >140 g/week

The prevalence of sarcopenia using the seven different diagnostic criteria is shown in Table 3. In men, the prevalence ranged from 0% to 20.8% in the lowest age category, from 0% to 31.2% in the middle age category and from 0% to 45.2% in the highest age category. In women, percentages ranged from 0% to 15.6%, 0% to 21.8% and 0% to 25.8% in the lowest, middle and highest age category, respectively.

**Table 3:** Prevalence of sarcopenia (*n* %) in the middle aged study population stratified by gender and age

Code^a^	Men				Women			
	Age (years)			Total	Age (years)			Total
	≤59	60–69	≥70		≤59	60–69	≥70	
	(*n* = 77)	(*n* = 186)	(*n* = 62)	(*n* = 325)	(*n* = 128)	(*n* = 170)	(*n* = 31)	(*n* = 329)
A	3 (3.9)	8 (4.3)	4 (6.5)	15 (4.6)	2 (1.6)	5 (2.9)	0	7 (2.1)
B	3 (3.9)	8 (4.3)	4 (6.5)	15 (4.6)	4 (3.1)	6 (3.5)	0	10 (3.0)
C	0	0	0	0	0	0	0	0
D	12 (15.6)	35 (18.8)	18 (29.0)	65 (20.0)	20 (15.6)	37 (21.8)	8 (25.8)	65 (19.8)
E1	3 (3.9)	17 (9.1)	16 (25.8)	36 (11.1)	4 (3.1)	8 (4.7)	4 (12.9)	16 (4.9)
E2	1 (1.3)	0	1 (1.6)	2 (0.6)	0	0	1 (3.2)	1 (0.3)
F1	16 (20.8)	58 (31.2)	28 (45.2)	102 (31.4)	0	0	1 (3.2)	1 (0.3)
F2	0	0	1 (1.6)	1 (0.3)	0	0	0	0
G	1 (1.3)	2 (1.1)	5 (8.1)	8 (2.5)	3 (2.3)	6 (3.5)	0	9 (2.7)

^a^The letters represent codes for the applied definition. The code is fully described in Table 1

Definitions A, B and C are based on the same formula taking height into account, but each comprised different reference populations and strategies to define cut-off points. In men, prevalence varied from 0% to 3.9% (lowest age category), 0% to 4.3% (middle age category) and 0% to 6.5% (highest age category). In women, this variation for definitions A, B and C was 0% to 3.1% (lowest age category), 0% to 3.5% (middle age category) and 0% (highest age category). When applying cut-off points for definition E class II, two men (0.6%) and one woman (0.3%) were classified as sarcopenic. The prevalence of sarcopenia was higher when applying cut-off points for definition E class I. Only one of the men (0.3%) was sarcopenic according to definition F class II criteria, based on muscle mass and height. The prevalence was higher using definition F class I criteria. The use of definition G, which included hand grip strength, gave a prevalence of less than 4% in this middle aged cohort.

**Fig. 1:**
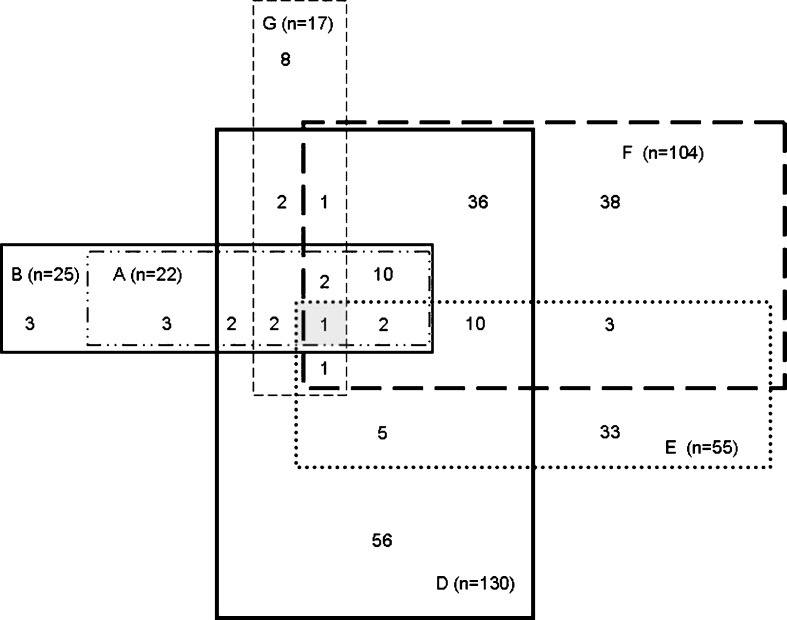
Number of participants identified as having sarcopenia according to various definitions, represented by letter codes. A description of the codes can be found in Table 1. A total of 654 were evaluated. Definition C, in which no participants were sarcopenic, is not shown. In definition E and F, class I and II sarcopenia are combined. Two subjects, one in whom sarcopenia was diagnosed according to F, G and E, and one in whom sarcopenia was diagnosed according to B and D, are not shown in the figure

Figure 1 shows the distribution of participants identified as sarcopenic according to the different diagnostic criteria. Definition C, which gave zero prevalence of sarcopenia, is omitted. For definition E and F, class I and class II sarcopenia are combined. Out of the 654 participants, 436 did not have sarcopenia according to any definition. For 218 participants, the diagnosis of sarcopenia depended on the diagnostic criteria applied. Only one of the participants (0.2%) was sarcopenic according to all six definitions.

Since the highest number of participants was sarcopenic according to definition D (*n* = 130) or F (*n* = 104), we analysed participant characteristics in these subgroups. We were mainly interested in the subgroups of 56 participants being sarcopenic only in definition D and of 38 participants being sarcopenic only in definition F. While BMI was comparable in these subgroups (in D 24.4 kg/m^2^ and in F 24.6 kg/m^2^), fat percentage was significantly higher in definition D compared to F (35.6% and 23.3%, respectively, *p* < 0.001).

## Discussion

In this large middle aged Dutch cohort, the prevalence of sarcopenia varied widely depending on which diagnostic criteria were used. Criteria based on low grip strength and skeletal lean mass failed to match with criteria based on appendicular lean mass. There was substantial overlap between diagnostic criteria A and B which are both based on the amount of appendicular lean mass, yet another cut-off point for the amount of appendicular lean mass resulted in the absence of sarcopenia (diagnostic criterion C). These findings clearly demonstrate the highly different selection of participants with the diagnosis sarcopenia using various criteria. Consequently, there are concerns about the validity of comparisons between studies using different criteria to diagnose sarcopenia.

The question arises which properties of skeletal muscle are represented by the term sarcopenia. Besides the production of force, muscle tissue is also an important regulator of biological processes. For instance, as a protein store it provides a homeostatic reserve to recover from disease (Englesbe et al. 2010). Furthermore, skeletal muscle has been identified as the major tissue involved in glucose metabolism (Corcoran et al. 2007; Shulman et al. 1990). Current evidence suggests that lean body mass may be a better measure for normalising dosages of drugs that are distributed and metabolised in lean tissue, compared with body surface area alone (Morgan and Bray 1994; Nawaratne et al. 1998; Prado et al. 2009). This underlines the importance to evaluate muscle mass in aging subjects.

In the present study, there was little overlap between individuals with low grip strength and low muscle mass using the diagnostic criteria. A possible explanation is that muscle strength is not only determined by muscle mass. The amount of muscle mass represents the number of sarcomeres that are in parallel. As each sarcomere is capable of exerting an amount of force, the number of sarcomeres in parallel, together with the quality of proteins and connective tissue determines the amount of force that a muscle can potentially exert. Additionally, muscle function is dictated by energy supply and neural control. Consequently, the terms muscle mass and muscle strength cannot be used interchangeably (Clark and Manini 2008). The rate of decline in muscle strength at older age appears to be higher than the decline in muscle mass in a 3-year longitudinal study, suggesting that factors other than muscle mass are influential (Goodpaster et al. 2006). Additionally, the increase of strength after resistance training is higher than the increase in muscle mass in older adults (Fiatarone et al. 1994; Kemmler et al. 2010; Orsatti et al. 2008; Sillanpaa et al. 2009). In a recent meta-analysis, it was shown that handgrip strength decreases each year with 0.37 kg (95% CI 0.31–0.44) after 50 years of age (Beenakker et al. 2010). Still, the low prevalence of sarcopenia in our cohort based on cut-off points for handgrip strength can possibly be explained by the fact that our cohort was middle aged and not over 80 years of age, where lower muscle strength becomes even more apparent (McNeil et al. 2005). Recently, a new term, dynapenia, has been developed to describe low muscle strength at old age (Clark and Manini 2008). The use of the terms dynapenia for low muscle strength, and sarcopenia for low muscle mass, emphasises the differences between muscle strength and mass. However, this terminology might overcomplicate the situation unnecessarily since muscle mass is also needed to generate strength. In other words, low muscle strength is one of the possible consequences of low muscle mass.

The prevalence of sarcopenia determined by diagnostic criteria using ALM corrected for height was not related to chronological age in women. However, the total lean mass as a percentage of total body mass was lower in all older subjects. This provides evidence that the interpretation of the amount of muscle mass is highly dependent on applied correction factors, such as fat mass and height. This is supported by the subgroup analysis of participants who were only sarcopenic in diagnostic criterium D, who had a high fat percentage and a relatively low muscle mass that was not recognised in diagnostic criteria adjusting for height only. Newman et al. found that correction for height only could lead to an overestimation of sarcopenia in underweight individuals, compared to an underestimation of sarcopenia in obese individuals (Newman et al. 2003). Furthermore, recent studies suggest that high fat mass is an important and independent determinant of functional status in elderly, even after adjustment for the level of physical activity (Lebrun et al. 2006; Rolland et al. 2009; Visser et al. 1998, 2005).

Next to a valid assessment of the amount of muscle mass, some variability emerges from the comparison to different reference populations and different cut-off points. Even with this variability, the degree of agreement between diagnostic criteria with the same formula but different cut-off points (definition A and B) was substantial. The prevalence of sarcopenia of zero percent using definition C can be explained by differences in reference populations. Furthermore, the prevalence of sarcopenia with definition A in this study cohort was much lower than reported in the same age categories by Baumgartner et al. (1998). In that study, using the same cut-off points in non-Hispanic whites, the prevalence of sarcopenia was found to be 13.5–23.1% below 70 years, and 19.8–33.3% between 70 and 74 years, in men and women, respectively. Differences in reference groups may be caused by age, ethnicity, genetic background and environmental factors such as the level of physical activity. Therefore, it is important to agree on reference populations that can be used in specific ethnic groups. Establishing reference databases generally requires a large sample size to achieve reliable results. Reference databases established to diagnose osteoporosis could function as a role model. Furthermore, it remains important to invest in longitudinal studies including the general population assessing the relation between muscle mass and functional outcomes to establish possible critical thresholds of muscle mass needed for muscle function.

Until now, attempts to approve on a consensus definition for sarcopenia failed. To the best of our knowledge, three international consortia have agreed on distinct definitions, which have not been generally accepted in the medical community. The first consensus definition was published by the Special Interest Groups (SIG) in 2009 (Muscaritoli et al. 2010). Here, the diagnosis sarcopenia is based on a combination of low muscle mass as defined by Janssen in 2002 (definition E) (Janssen et al. 2002), together with low gait speed, which is walking speed below 0.8 m/s in the 4-m walking test, or another functional test (Muscaritoli et al. 2010). The second consensus definition was published by The European Working Group on Sarcopenia in Older People (EWGSOP) in 2010. The EWGSOP included a degree of severity of sarcopenia in the definition. ‘Presarcopenia’ was defined as low muscle mass, ‘sarcopenia’ as low muscle mass together with either low muscle strength or performance and ‘severe sarcopenia’ as a combination of all three. In addition, it was proposed that sarcopenia should be considered ‘primary’ when no other cause is evident but ageing itself, and ‘secondary’ when one or more other causes are evident (Cruz-Jentoft et al. 2010). This terminology is not acceptable in modern gerontology, as age in itself is no longer considered a causal factor of disease (Holliday 1999). In the third consensus definition, Fielding et al. based the diagnosis of sarcopenia on low muscle mass as defined by Delmonico et al. (definition B) (Delmonico et al. 2007), together with low gait speed defined as less than 1 m/s (Fielding et al. 2011).

In our opinion, these consensus definitions are not clinically applicable. First of all, using the SIG definition, none of the participants in the present study fulfilled the criteria for low muscle mass. Furthermore, gait speed is not a parameter of muscle function alone, but also dependent on other factors such as cognition, neural control, joint function and cardiovascular fitness (Hajjar et al. 2011). The EWGSOP definition lists different ways to diagnose sarcopenia, without making a choice which measurement should be used. Consequently, the huge variability of prevalence of sarcopenia highlighted in the present study would therefore still be present. It is important to keep the differences between diagnostic criteria in consideration when interpreting the results of studies where muscle tissue is evaluated.

The strength of the present study is that the currently used diagnostic criteria of sarcopenia were applied to one study population. There are some limitations to the use of DXA since different densitometers and software versions have been shown to give different estimates of body composition (Paton et al. 1995; Tataranni et al. 1996). The use of different equations between various BIA machines could also contribute to different body composition parameters. Abnormal hydration status or extreme body weight might also influence the measurements (Woodrow 2009). Previously, we have shown an excellent correlation between BIA and DXA measurements (Ling et al. 2011a); therefore, we were able to apply diagnostic criteria based on both DXA and BIA measurements. A possible limitation of this study is selection bias of the participants since only the partners of the offspring of nonagenarian siblings are considered to be representatives of the general population. However, the offspring of long-lived families did not differ significantly from their partners in fat percentage, muscle mass and handgrip strength (Ling et al. 2011b). Therefore, this would not influence conclusions made in the present study. Moreover, participants were middle aged; the degree of agreement between the different diagnostic criteria for sarcopenia might be different in an oldest old population. Another limitation is that we had no functional outcome measures available for participants in this study. We were not able to apply diagnostic criteria for sarcopenia based on gait speed.

In conclusion, the prevalence of sarcopenia varies widely depending on the applied diagnostic criteria. A consensus definition is necessary in order to make studies comparable and for implementation in clinical care. Until there is universal consensus, the diagnostic criteria used to define sarcopenia in separate studies should be clearly described, without the interchangeable use of the terms muscle mass and muscle strength. Further research should focus on establishing an appropriate formula to correct the amount of muscle mass for factors such as height and fat mass, and take into account differences in ethnicity when subjects are compared to reference populations.
